# Obtainment of Two Monomorphic Nematocysts from *Nemopilema nomurai* (Cnidaria: Scyphozoa) and Comparative Analysis of the Biological Activities of Their Contents

**DOI:** 10.3390/md23110421

**Published:** 2025-10-30

**Authors:** Yongfei Lyu, Yichao Huang, Juxingsi Song, Dayuan Zhou, Shuaijun Zou, Jie Li, Fan Wang, Qianqian Wang, Yanan Hu, Shaoqian Zhu, Sai Luo, Xinyue Gan, Liming Zhang, Guoyan Liu

**Affiliations:** Naval Special Medical Center, Naval Medical University, Shanghai 200433, China; lvyongfei2022@163.com (Y.L.); 18065615825@163.com (Y.H.); song9935@163.com (J.S.); 13201555836@163.com (D.Z.); sjzou@smmu.edu.cn (S.Z.); lijie1992@smmu.edu.cn (J.L.); fanwang1130@126.com (F.W.); abc_w@smmu.edu.cn (Q.W.); yananhu20192022@163.com (Y.H.); shaoqianzhu2018@163.com (S.Z.); 15056344020@163.com (S.L.); ganxinyue@shu.edu.cn (X.G.)

**Keywords:** jellyfish, *Nemopilema nomurai*, monomorphic nematocysts, isolation and purification, biological activity, toxicity

## Abstract

*Nemopilema nomurai* is a species of common large toxic jellyfish in China seas, and its tentacle tissues contain various types of nematocysts. However, the correlation between the morphology and function of nematocysts still remains unclear. In this study, we first obtained two monomorphic nematocysts with high-purity from *N. nomurai*, namely Anisorhizas and O-isorhizas, by density gradient centrifugation: the Anisorhizas is small and rod-shaped and the O-isorhizas is larger and spherical. Upon deionized water stimulation, O-isorhizas exhibited a stronger discharge capability than Anisorhizas. The nematocyst contents of Anisorhizas (AnC) and O-isorhizas (OnC) were extracted separately, and their composition and bioactivities were analyzed simultaneously. The protein bands by SDS-PAGE revealed similar distributions in AnC and OnC, except that the protein band distribution in OnC was more extensive. OnC showed stronger cytotoxicity, hemolytic activity, metalloprotease activity, and serine protease activity than AnC. In contrast, AnC exhibited a higher antioxidant activity and significant proinflammatory activity. Both AnC and OnC exhibited antimicrobial activities against certain marine pathogenic Vibrios. These results suggest that O-isorhizas, with the larger capsule capability, stronger discharge ability and toxicity, likely plays a major role in inducing toxic effects and tissue damage, while Anisorhizas, being smaller and less toxic, may undertake preferentially other functions, such as synergistic predation, environmental stress adaptation, and energy balance maintenance. This study provides insights into the morpho-functional relationship between various types of nematocysts, and also lays a foundation for further exploration of the functional diversity of nematocysts and the mechanisms underlying jellyfish envenomation.

## 1. Introduction

Jellyfish, the representative of cnidarians among plankton, are characterized by their remarkable species diversity and wide geographical distribution. As one of the most common venomous marine organisms, jellyfish stings can induce a broad spectrum of clinical symptoms, including localized pain, pruritus, erythema and edema. In severe cases, there may even be limb swelling, tachypnea, cardiac arrest and even death [1,2,3]. Studies have shown that jellyfish toxins exhibit diverse toxic effects including neurotoxicity, myotoxicity, hemolytic activity, and a wide range of other biological activities [4,5,6,7,8,9,10]. However, due to the instability of jellyfish toxins [11,12,13], the research on jellyfish venom obviously lags behind that on the venoms derived from other common toxic animals, and its precise composition and mechanisms of action remain still unclear [14].

Nematocysts, featuring robust capsule walls, are a type of sac-like organelles unique to cnidarians. They integrate sensory and secretory functions while playing pivotal roles in predation, defense, and locomotion [15,16]. Nematocysts are located within the densely packed cnidocytes existing in jellyfish tentacles. When the tentacles perceive physical contact (pressure) or detect specific chemical signals, the discharges of nematocysts are triggered. The osmotic pressures inside and outside the capsule change drastically. The capsule lid pops open, and the thread is ejected at an extremely high acceleration [17]. Its sharp tip can quickly penetrate the stratum corneum of the skin or the prey’s body surface barrier, and the venom is immediately injected under high pressure to complete the envenomation [18]. Nematocysts, as highly specialized structures, exhibit great diversity in morphology and size, which is closely related to many aspects such as the survival and functional adaptation of cnidarians [19]. For example, the size of nematocysts often matches the type of prey they target. Nematocysts with larger sizes usually have longer and stronger spreads, which enable them to ensure the effective delivery of venom when penetrating larger prey or thicker tissues. In addition, the size, shape and distribution of nematocysts are one of the key features for identifying cnidarians in taxonomy [20].

To date, more than 30 types of nematocysts have been identified in cnidarians [20,21], which can be functionally classified into four categories [19]. The first category is responsible for prey immobilization; the second category facilitates prey penetration while simultaneously injecting venom-these two categories of nematocysts cooperate to complete the predation process and can be collectively referred to as predatory nematocysts [22]; the third category is associated with locomotion, enabling adhesion to other marine organisms or substrate surfaces to assist movement; and the fourth category serves defensive purposes. Based on the morphological classification [23,24], nematocysts can be divided into open-thread type and closed-thread type according to whether the top of thread is open. The open-thread type can be further categorized into nematocysts with a central axis thread and those without a central axis thread. The former includes euryteles, mastigophores, stenoteles, etc., and the latter includes isorhizas (such as O-isorhizas, etc.) and Anisorhizas, etc.

The nematocysts of jellyfish exhibit remarkable diversity according to their morphological characteristics and biological functions, and their venomous contents are highly complex, predominantly comprising protein/peptide toxins [25,26]. However, the current research on the components and effects of monomorphic nematocysts remain relatively limited, with most investigations focusing on predatory nematocysts (penetrating/venom-injecting types) and their discharge mechanisms and toxic effects. For example, Brinkman et al. [27] successfully isolated and purified two distinct types of nematocysts, mastigophores and a mixed sample of isorhizas with trirhopaloids, from *Chironex fleckeri*. Through integrated transcriptomics and proteomic analysis of their contents, the study revealed significant compositional and functional relationships between these nematocyst subtypes. Notably, comparative analysis demonstrated minimal differences in the toxin profiles, providing novel insights into potential synergistic mechanisms underlying jellyfish nematocyst function. Wang et al. [14] isolated and purified predatory nematocysts, namely isorhizas and mastigophores from *Cyanea capillata* and *N. nomurai*, respectively. Through combined transcriptomic and proteomic analysis of their contents, the compositional differences between these predatory nematocysts were elucidated, thereby providing a molecular basis for understanding the underlying mechanisms of distinct jellyfish sting symptoms and developing targeted treatments. Nevertheless, except for predatory nematocysts, the research on other types of nematocysts is still largely confined to descriptive level, with their composition characteristics, bioactivities and functions remaining poorly characterized. Therefore, the isolation and purification of monomorphic nematocysts is of great importance for gaining deeper insights into the functional diversity of jellyfish nematocysts and advancing the prevention and treatment of jellyfish sting.

*N. nomurai* is a large toxic jellyfish commonly found in China seas, with a wide distribution and medical importance [1,2,3,28,29]. Current research on *N. nomurai* venom primarily adopts the method of direct extraction from the tentacles [14,27,30,31,32,33,34,35,36,37], which fails to elucidate specific nematocyst functions. The obtainment of monomorphic nematocysts enables precise characterization of specific nematocyst function while minimizing the variability between sample batches during toxin research. In this study, we isolated two monomorphic nematocysts from *N. nomurai* tentacles, characterized their microstructures and discharge capacities, and subsequently analyzed the toxic effects and bioactivities of their contents to explore the potential relationships between the morphological structures and biological functions thereby providing a deeper understanding of jellyfish nematocysts and their functional diversity.

## 2. Results

### 2.1. Microscopic Morphology of N. nomurai Nematocysts

**Figure 1:**
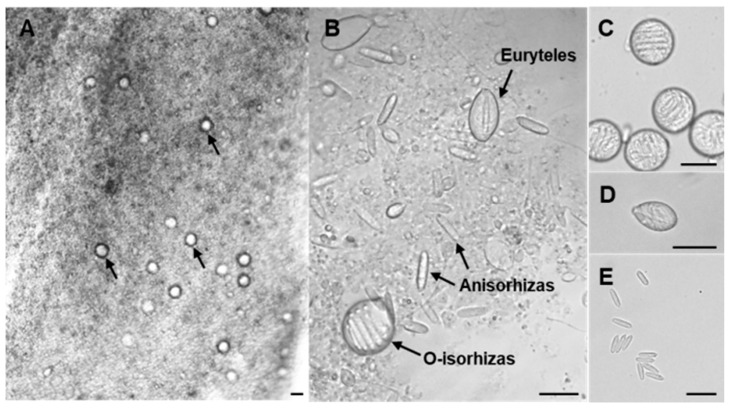
Light microscopic observation of nematocysts in *N. nomurai* tentacles. (**A**) Microscopic image of jellyfish tentacle (magnification 10×). Arrows indicate O-isorhizas. (**B**) Three types of nematocysts observed in the tentacles (magnification 40×). (**C**) O-isorhizas. (**D**) Euryteles. (**E**) Anisorhizas. Bar scale, 20 μm.

Through light microscopic observation of *N. nomurai* tentacles, we identified three distinct types of nematocysts. In accordance with established nematocyst classification systems [23,24], they were correspondingly categorized as Anisorhizas, O-isorhizas, and Euryteles (Figure 1).

Initial observation at 10× magnification revealed O-isorhizas nematocysts (Figure 1A, arrowhead), while all three types became clearly distinguishable at 40× magnification (Figure 1B). O-isorhizas, being the largest among the three types, appear circular or possess oval morphology with observable coiled structures of threads in the nematocysts (Figure 1C). Anisorhizas, which were the smallest and most numerous, exhibited a rod-shaped morphology, though their internal structures remained poorly defined under microscopic examination (Figure 1E). Euryteles, present in relatively fewer numbers, demonstrated spindle-shaped containing prominent axial structures surrounded by coiled spreads (Figure 1D).

**Figure 2:**
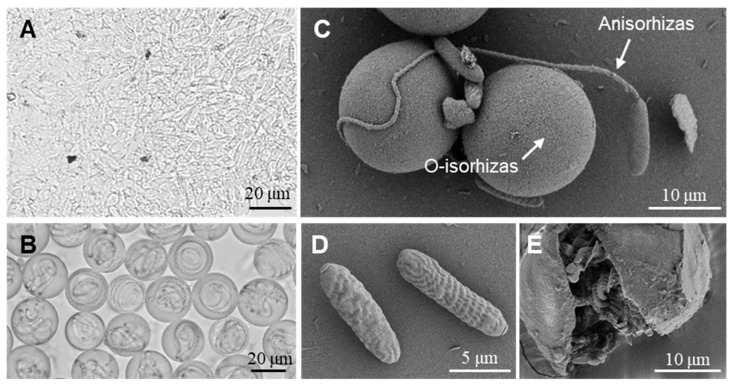
Microscopic morphology of Anisorhizas and O-isorhizas. (**A**) Anisorhizas observed under light microscope (40× magnification). (**B**) O-isorhizas observed under light microscope (40× magnification). (**C**) O-isorhizas (undischarged) and Anisorhizas (discharged) observed under SEM, respectively. (**D**) Anisorhizas (undischarged) observed under SEM. (**E**) Ruptured O-isorhizas observed under SEM.

With Percoll solution-based density gradient centrifugation, we established a method for isolation of monomorphic nematocysts by optimizing conditions such as the Percoll concentration gradient and centrifugation parameters (see Section 4.3). Through this method, high-purity Anisorhizas and O-isorhizas nematocysts were successfully obtained, which were subsequently examined by light microscopy and scanning electron microscopy (SEM) (Figure 2).

Light microscopic observation revealed that Anisorhizas were relatively small with a rod-shaped morphology, measuring approximately 2 μm in the short axis and 10–15 μm in the long axis. However, their internal structures remained indistinct under light microscopy (Figure 2A). In contrast, O-isorhizas appeared circular or oval in shape with a diameter of about 20 μm, displaying clearly visible coiled spread within the capsules (Figure 2B). SEM examination demonstrated significant size differences between these two nematocyst types. Undischarged O-isorhizas exhibited spherical morphology with smooth surfaces, while discharged Anisorhizas showed spreads extending several times longer than the capsule itself (Figure 2C). Undischarged Anisorhizas maintained their characteristic rod-shaped appearance (Figure 2D). Figure 2E presents a ruptured O-isorhizas, revealing the distinct spiral structure of the spread inside.

### 2.2. Discharge Abilities of Anisorhizas and O-Isorhizas

**Figure 3:**
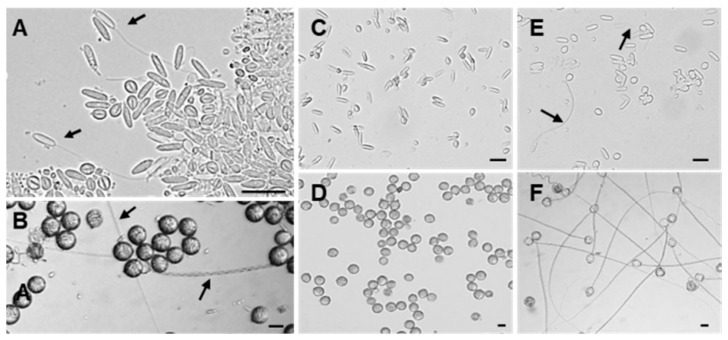
Discharge abilities of Anisorhizas and O-isorhizas. (**A**) Discharged Anisorhizas and (**B**) discharged O-isorhizas were observed during isolation. (**C**) Undischarged Anisorhizas and (**E**) undischarged O-isorhizas were observed before stimulation with deionized water. (**D**) Discharged Anisorhizas and (**F**) discharged O-isorhizas were observed after treatment with deionized water. The arrows indicate the discharged nematocysts. Bar scale, 20 μm.

During the isolation process of the two types of nematocysts, we found that some nematocysts had been in a discharged state (Figure 3A,B). To clarify the discharge abilities of O-isorhizas and Anisorhizas, the responses of the nematocysts following stimulation with deionized water were examined. Before stimulation, both types of nematocysts were evenly distributed and remained in an undischarged state (Figure 3C,D). After stimulation with deionized water, under the microscope (40× magnification), only a small number of Anisorhizas nematocysts discharged (Figure 3E), whereas the majority of O-isorhizas exhibited complete discharge (Figure 3F). These results demonstrate that both types of nematocysts can be stimulated to discharge through osmotic pressure changes within their capsules. Under deionized water stimulation, O-isorhizas displayed significantly greater discharge ability compared to Anisorhizas.

### 2.3. Protein Composition in Nematocysts

**Figure 4:**
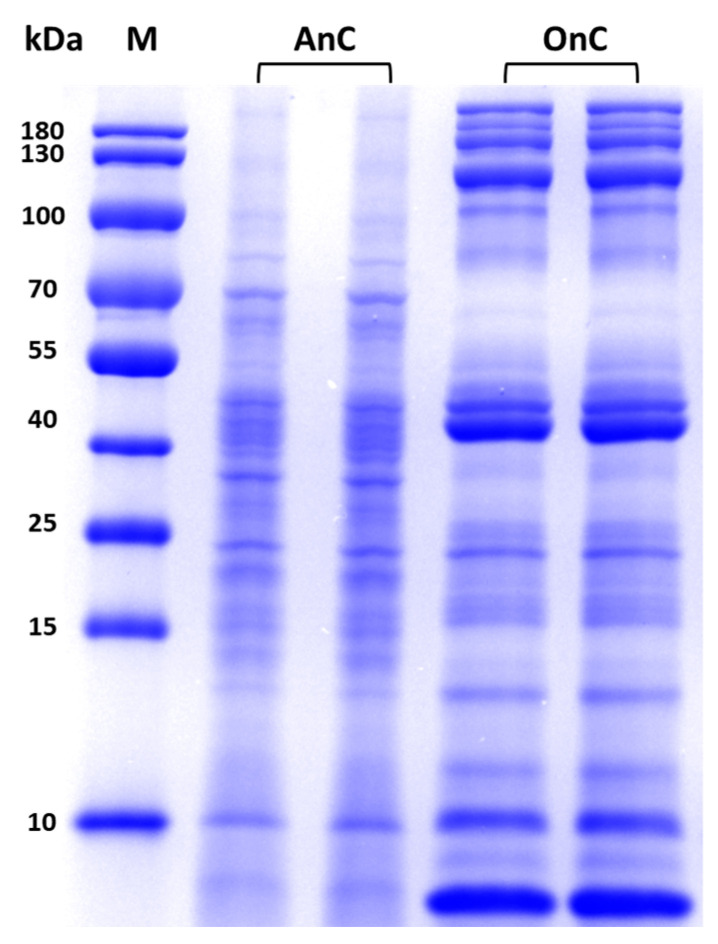
SDS-PAGE analysis of AnC and OnC. AnC and OnC (10 μg of total protein) were separated in a 12% polyacrylamide gel and stained with Coomassie Brilliant Blue. M = markers, and the molecular masses of the protein standards are shown in kilodaltons (kDa).

Currently, the extraction of nematocyst contents commonly employs solutions such as Tris-HCl buffer or physiological saline, combined with mechanical disruption methods including grinding, homogenization, or ultrasonication. In this study, ultrasonication was used to extract the contents of O-isorhizas and Anisorhizas, defined as O-isorhizas nematocyst content (OnC, total protein amount 8.4 mg; 1.4 mg protein/kg tentacle tissue) and Anisorhizas nematocyst content (AnC, total protein amount 3.3 mg; 0.55 mg protein/kg tentacle tissue), respectively. The protein concentrations of the stock solutions were determined to be 2.88 mg/mL for OnC and 1.32 mg/mL for AnC, respectively. SDS-PAGE analysis revealed that AnC proteins were predominantly distributed within the 10~100 kDa range, with obvious bands observed at ~10 kDa and 40~100 kDa. In contrast, OnC exhibited a broader distribution, spanning 10~180 kDa, with prominent bands within ~10 kDa, 40~55 kDa, and 100~180 kDa range (Figure 4). These results indicated that the protein compositions of AnC and OnC were generally similar, while there were differences in some aspects, suggesting potential functional specificity of Anisorhizas and O-isorhizas.

### 2.4. Cytotoxicity of AnC and OnC

**Figure 5:**
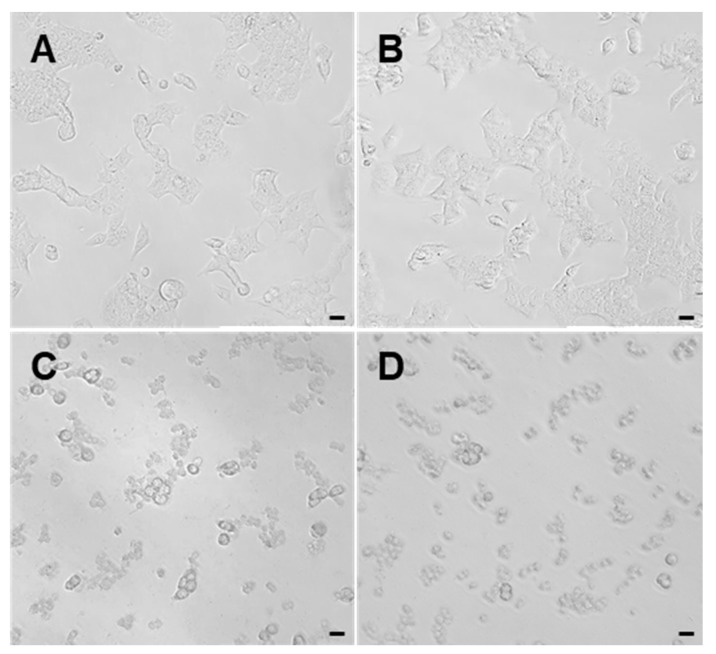
Morphological characterization of HCT116 cells after treatment with AnC, OnC and TeC. HCT116 cell suspension was seeded in a 96-well plate and incubated in an incubator for 24 h. The medium was discarded, 100 μL of (**A**) PBS and 0.5 μg/mL of (**B**) AnC, (**C**) OnC, and (**D**) TeC were added, respectively, and the incubation continued for 4 h. The morphology of the cells was observed under a microscope. Bar scale, 20 μm.

The extract of *N. nomurai* tentacles contains diverse nematocyst types and has been demonstrated to possess multiple biological activities. In this study, we used tentacle extract content (TeC) from *N. nomurai* as a control to analyze the toxic effects of AnC and OnC. HCT116 cells were treated with 0.5 µg/mL AnC, OnC, and TeC for 4 h, respectively. AnC-treated cells were closely arranged without overlapping, displaying polygonal or spindle-shaped morphology with clear edges, transparent cytoplasm and excellent light refractivity (Figure 5B), showing no obvious difference compared with the untreated group (Figure 5A). In contrast, OnC and TeC treatment induced distinct cytotoxicity, characterized by loss of membrane integrity in most cells, granular appearance, and transition to rounded/elliptical morphology with reduced adhesion capacity (Figure 5C,D). These results indicated that AnC did not exhibit obvious cytotoxicity to HCT116 cells, while OnC and TeC showed significant cytotoxicity.

**Figure 6:**
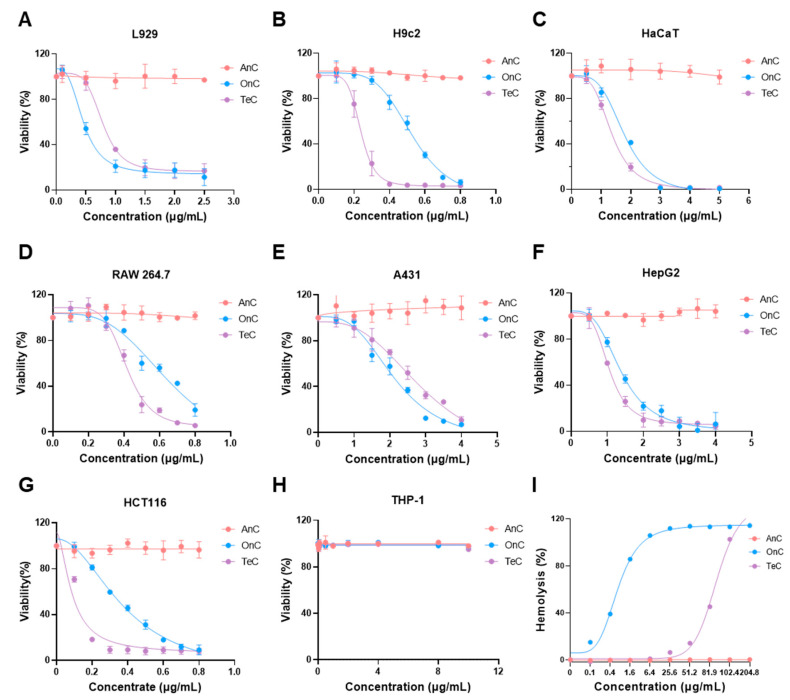
Cytotoxicity and hemolytic activity of AnC, OnC and TeC. The Cell Counting Kit-8 assay was used to detect the cytotoxicity of AnC, OnC and TeC to different cell lines, including 3 normal cell lines such as (**A**) L929, (**B**) H9c2 and (**C**) HaCaT, and 5 tumor cell lines such as (**D**) RAW 264.7, (**E**) A431, (**F**) HepG2, (**G**) HCT116 and (**H**) THP-1. (**I**) Mouse erythrocytes were used to measure hemolytic effect of AnC, OnC and TeC. The results are expressed as the means ± SD of three independent experiments.

Further cytotoxicity evaluation using CCK-8 assays across multiple cell lines revealed different toxicity profiles (Figure 6). Both OnC and TeC demonstrated potent cytotoxicity toward the three detected normal cell lines (L929, H9c2, HaCaT), with viability decreasing concentration-dependently. However, AnC had little impact on the survival rate of these cells at the same concentration and did not show obvious cytotoxicity (Figure 6A–C). When tested in tumor cell lines (A431, HepG2, HCT116, THP-1, RAW 264.7), OnC and TeC exhibited significant toxicity toward all tested cells except THP-1, and the cell survival rate decreased significantly with the increase in concentrations of OnC and TeC. In contrast, AnC did not show significant cytotoxicity against tumor cells within this concentration range (Figure 6D–H).

### 2.5. Hemolytic Effects of AnC and OnC

As shown in Figure 6I, both OnC and TeC exhibited significant concentration-dependent hemolytic effects against murine erythrocytes. The half maximal hemolytic concentration (HC_50_) values for OnC and TeC were 2.91 µg/mL and 81.2 µg/mL, respectively. The complete hemolysis concentrations for OnC and TeC were 25.6 µg/mL and 102.4 µg/mL, respectively. Notably, no detectable hemolytic effect was observed for AnC within the tested concentration range.

### 2.6. Enzyme Activity of AnC and OnC

#### 2.6.1. Protease Activity

**Figure 7:**
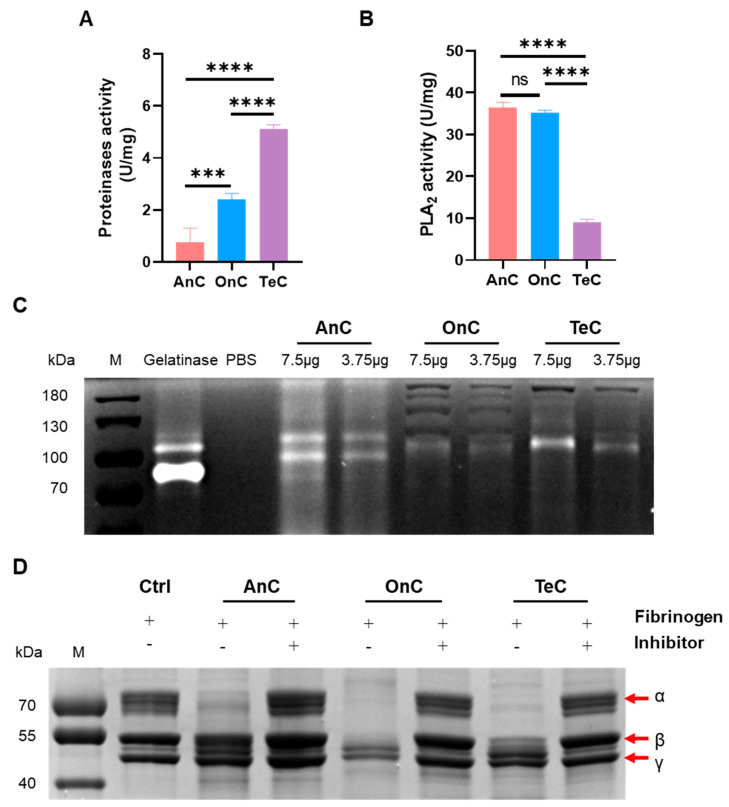
Enzyme activity of AnC, OnC and TeC. (**A**) Azocasein degradation assay (U/mg, mean ± SD, *n* = 3). One unit of activity was defined as the amount of enzyme required to cause an increase in OD by 0.01 at 450 nm. (**B**) PLA_2_ activity of AnC, OnC and TeC (U/mg, mean ± SD, *n* = 3). A change in absorbance of 0.10 AU at 425 nm was equivalent to the release of 25.8 nanomoles of chromophore (3-hydroxy-4-nitrobenzoic acid). (**C**) Gelatin enzyme assay. (**D**) Serine proteases activity of AnC, OnC and TeC. M = markers. (ns: *p* > 0.05, *** *p* < 0.001, **** *p* < 0.0001).

Azocasein degradation assays revealed significant protease activities in AnC, OnC, and TeC, with enzyme vitality of 0.80 ± 0.40 U/mg, 3.43 ± 0.25 U/mg, and 5.8 ± 0.51 U/mg, respectively (Figure 7A). It is worth noting that OnC exhibited 4.29-fold higher protease activity than AnC.

Gelatin zymography analysis demonstrated that gelatinase (positive control) displayed distinct lytic bands at 72~92 kDa and ~100 kDa, showing gelatinolytic activity (Figure 7C). The bands corresponding to 72 kDa and 92 kDa, respectively, suggested the potential presences of pro-MMP-2 and pro-MMP-9 in the sample. Similarly to the gelatinase group, AnC produced two concentration-dependent lytic bands near 72 kDa and 92 kDa, indicating that it may contain pro-MMP-2 and pro-MMP-9. Notably, AnC exhibited an additional band at ~110 kDa, while OnC and TeC showed prominent bands at ~100 kDa, respectively. These bands may correspond to the glycosylated MMP-9 forms [38,39]. To sum up, these results indicate that both AnC and OnC have gelatinolytic activity.

#### 2.6.2. Phospholipase A_2_ (PLA_2_) Activity

NOBA is a synthetic chromogenic substrate, which releases p-nitrophenol (pNP) with strong absorbance at 410 nm upon phospholipase A_2_ (PLA_2_)-mediated hydrolysis. As shown in Figure 7B, AnC, OnC and TeC can all catalyze the hydrolysis of NOBA, indicating significant PLA_2_ activity. AnC and OnC showed comparable activities (36.38 ± 1.37 U/mg and 35.17 ± 0.65 U/mg, respectively), approximately 3.98- and 3.85-fold higher than TeC (9.12 ± 0.65 U/mg).

#### 2.6.3. Serine Protease Activity

Serine proteases (e.g., thrombin, plasmin, and bacterial proteases) specifically degrade the nature substrate fibrinogen, generating fibrin monomers or small peptide fragments. Fibrinogen degradation assays (Figure 7D) demonstrated that the substrate fibrinogen (as the control) showed bands near ~70 kDa, ~55 kDa and ~50 kDa, corresponding to the α-, β- and γ-chain, respectively. Under the action of OnC, the α- and β-chains of fibrinogen were almost completely degraded, and the γ-chain was partially degraded (indicated by reduced band intensity), exhibiting the most potent fibrinogen degradation activity. TeC completely degraded α-chain while partially cleaving β- and γ-chains. In contrast, under the action of AnC, most of the α-chain was degraded without detectable degradation of β- or γ-chains. The fibrinogenolytic activities were all significantly inhibited by 1,10-phenanthroline. These results indicate that AnC, OnC and TeC have distinct serine protease activity, with the activity of OnC being the strongest, followed by TeC, and AnC being the weakest.

### 2.7. Antimicrobial Activity of AnC and OnC

Previous studies have shown that jellyfish venoms possess antimicrobial activities [40,41,42,43]. Therefore, in this study, we evaluated the antimicrobial activities of AnC, OnC, and TeC against four common terrestrial pathogenic bacteria and six marine pathogenic Vibrios.

**Figure 8:**
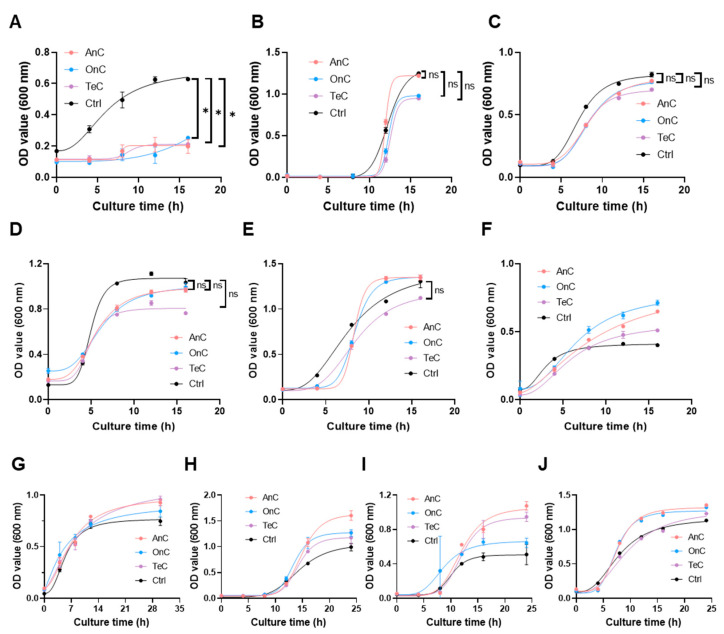
Inhibitory effects of AnC, OnC and TeC on the growth of common terrestrial pathogenic bacteria and pathogenic vibrios. (**A**) *Vibrio mimicus*. (**B**) *Pseudomonas aeruginosa*. (**C**) *Vibrio vulnificus*. (**D**) *Vibrio natriegens*. (**E**) *Vibrio parahaemolyticus*. (**F**) *Vibrio anguillarum*. (**G**) *Escherichia coli*. (**H**) *Staphylococcus aureus*. (**I**) *Bacillus subtilis*. (**J**) *Vibrio alginolyticu*. The asterisks indicate a significance difference from the control value, with ns *p* > 0.05, * *p* < 0.05.

As shown in Figure 8A, AnC (stock solution, 1.32 mg/mL), OnC (stock solution, 2.88 mg/mL) and TeC (stock solution, 2.53 mg/mL) exhibited significant growth inhibitory effects against *Vibrio mimicus* within 16 h, with inhibition rates of 68.58%, 59.81% and 66.40%, respectively. Notably, for the other nine pathogenic bacterial (*Pseudomonas aeruginosa*, *Vibrio vulnificus*, *Vibrio natriegens*, *Vibrio parahaemolyticus*, *Vibrio anguillarum*, *Bacillus subtilis*, *Escherichia coli*, *Vibrio alginolyticu*, and *Staphylococcus aureus*), AnC, OnC and TeC did not show obvious inhibitory effects within 16 h (Figure 8B–J). These results demonstrate that the antimicrobial effects of AnC and OnC may not be broad-spectrum. In the future, the range of tested pathogenic bacteria can be expanded to more comprehensively understand their antimicrobial spectrum.

### 2.8. Antioxidant Activity of AnC and OnC

**Figure 9:**
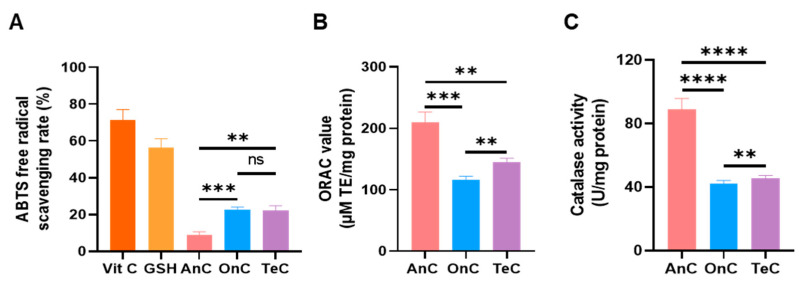
Antioxidant capacity of AnC, OnC and TeC. (**A**) ABTS^+^ scavenging capacity (T-AOC ABTS assay). (**B**) Oxygen radical scavenging capacity activity (ORAC). (**C**) Catalase activity. ns *p* > 0.05, ** *p* < 0.01, *** *p* < 0.001, **** *p* < 0.0001.

The ABTS^+^ radical scavenging capacities of 1 mg/mL AnC, OnC, and TeC were determined using the total antioxidant capacity (T-AOC) kit (Figure 9A). With vitamin C (1 mg/mL) and glutathione (GSH, 1 mg/mL) as the positive control, their scavenging rates were measured to be 71.56 ± 5.51% and 56.25 ± 5.08%, respectively. OnC and TeC exhibited comparable ABTS^+^ scavenging activities (22.86 ± 1.163% and 22.32 ± 2.488%, respectively), approximately 2-fold higher than AnC (9.00 ± 1.69%).

The oxygen radical absorbance capacity (ORAC) assays were used to determine the peroxyl radical scavenging capacities of AnC, OnC and TeC (Figure 9B). AnC had the strongest activity (209.7 ± 16.99 µM TE/mg protein), exceeding OnC (116.0 ± 6.07 µM TE/mg protein) and TeC (145.4 ± 6.11 µM TE/mg protein) by 1.80- and 1.44-fold, respectively.

The result of catalase activity detection (Figure 9C) showed that AnC possessed the strongest enzymatic activity (89.05 ± 6.81 U/mg protein), which was 2.09- and 1.95-fold higher than OnC (42.48 ± 1.80 U/mg protein) and TeC (45.74 ± 1.63 U/mg protein), respectively.

### 2.9. Proinflammatory Effects of AnC and OnC

Previous studies reported that low concentrations of jellyfish tentacle extracts exhibited proinflammatory properties [32,37,44,45], though no such investigations have been conducted on isolated monomorphic nematocyst extracts. Therefore, we further evaluated the proinflammatory potential of AnC and OnC.

**Figure 10:**
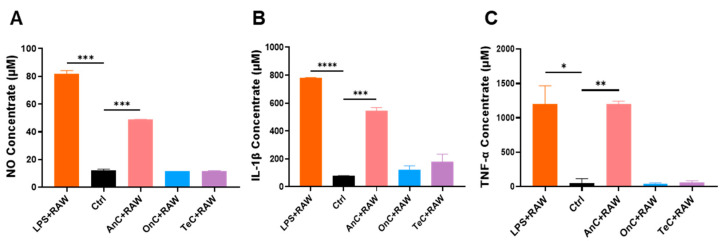
Proinflammatory effects of AnC, OnC and TeC. (**A**) The levels of NO, (**B**) The levels of IL-1β, (**C**) The levels of TNF-α. The asterisks indicate a significance difference from the control value, with * *p* < 0.05, ** *p* < 0.01, *** *p* < 0.001, **** *p* < 0.0001.

RAW 264.7 cells were treated with 0.1 μg/mL AnC, OnC, or TeC for 24 h, with untreated cells as blank control and LPS (1 μg/mL)-treated cells as positive control. As shown in Figure 10, LPS stimulation significantly increased the concentrations of nitric oxide (NO, 81.57 ± 2.598 μM), IL-1β (779.7 ± 3.152 μM), and TNF-α (1202 ± 264.0 μM) compared to blank controls. Notably, after AnC treatment, the concentrations of NO, IL-1β and TNF-α also increased significantly, being 48.71 ± 0.2886 μM, 547.6 ± 20.64 μM, and 1204 ± 38.18 μM, respectively. In contrast, neither OnC nor TeC treatment induced significant upregulation of these proinflammatory factors. These findings suggest that AnC contains substantial inflammatory components that may play a role in mediating inflammatory responses following jellyfish envenomation, while OnC appears to lack such activity. The molecular mechanisms underlying these effects remain to be further investigated.

## 3. Discussion

Jellyfish are common venomous marine organisms, characterized by their specialized nematocysts that serve as venom reservoirs for prey capture and defense. Compared to the toxic apparatuses of other venomous organisms, jellyfish nematocysts are diverse in types, presenting technical challenges for isolation due to their structural heterogeneity. A single nematocyst contains only a small amount of toxins, which is unstable and easy to inactivate [11,12]. These factors delayed research on jellyfish venoms. Therefore, the isolation of monomorphic nematocyst is a prerequisite for investigating nematocyst classification, functional characteristics, and venom composition. In this study, we established an optimized density gradient centrifugation protocol for efficient isolation of monomorphic nematocysts from *N. nomurai*, yielding two distinct types of nematocysts: O-isorhizas and Anisorhizas. The purified nematocysts demonstrated morphological integrity and functional viability, as confirmed by microscopic examination and discharge assays.

O-isorhizas and Anisorhizas displayed significant differences in physical parameters including volume and diameter, which makes them particularly amenable for isolation via density gradient centrifugation. Taking Anisorhizas as an example, the optimized isolation protocol involves sequential steps: initial primary separation through collection of tentacle autolysis filtrates via centrifugation, followed by density gradient optimization, which is the key to obtain high-purity monomorphic nematocysts. Through comparative evaluation of the media including sucrose, Ficoll, and Percoll, we identified Percoll as the optimal medium due to its low osmolarity and viscosity, rapid gradient formation, easy removability through washing, and exceptional stability against temperature and pH fluctuations. The purification stage employs a discontinuous gradient of Percoll (30%, 50%, 70%, 90%, respectively) with centrifugation at 150× *g* for 60 min to yield high-purity Anisorhizas, which are subsequently subjected to seawater washes (1000× *g*, 60 min) for complete medium removal. Through this optimized protocol, we successfully obtained a large amount of Anisorhizas nematocysts of high purity.

Microscopic observation revealed distinct morphological differences between the two nematocyst types. Compared with Anisorhizas, O-isorhizas has a larger volume, and the spread inside the capsule exhibits an obvious spiral structure. Both O-isorhizas and Anisorhizas could be induced to discharge through osmotic pressure changes, with O-isorhizas exhibiting greater discharge capability upon deionized water stimulation. These results indicate some correlation between the morphological structures and biological functions of nematocysts, suggesting that the spiral structure of the thread inside O-isorhizas and its large volume may endow O-isorhizas with superior discharge and penetrating abilities, enabling efficient accumulation and transfer of energy to promote rapid discharge of the thread, which can break through the defensive barriers of prey and natural enemies. Therefore, O-isorhizas likely serves as the primary offensive/defensive mechanism during jellyfish stinging. Although Anisorhizas demonstrate relatively weaker discharge ability compared to O-isorhizas, they appear to play a complementary role in the stinging process, potentially involved in coordinated venom delivery to enhance venom injecting effects and improve prey capture and defensive efficiency.

Through ultrasonication-mediated disruption, we successfully obtained the intracapsular contents from O-isorhizas and Anisorhizas. SDS-PAGE analysis revealed generally similar but distinct protein banding patterns between the two nematocysts, suggesting potential functional specialization. Previous studies have shown that the composition of jellyfish venom in nematocysts is complex, including both proteinaceous (up to 220 kDa) and non-proteinaceous components [46]. Notably, multiple enzymatic constituents have been identified in jellyfish venoms, with phospholipases, metalloproteinases, and serine proteases representing the most prominent components [47].

PLA_2_, a ubiquitous component of animal venoms, exerts hemolytic effects through hydrolysis of membrane phospholipids into lysophospholipids, thereby disrupting erythrocyte membrane integrity and directly causing hemolysis [48,49]. Wang et al. [14] isolated nematocysts (mastigophores) from jellyfish *Cyanea capillata* and determined the PLA_2_ activity of their contents (CnV) to be 338.3 ± 42.2 nmol/min/mg, which was approximately 7.04-fold and 3.34-fold higher than that of AnC (48.02 ± 1.37 nmol/min/mg, 36.38 ± 1.37 U/mg) and OnC (101.29 nmol/min/mg, 35.17 ± 0.65 U/mg), respectively. Phospholipase A_2_ is a typical toxin protein family. This result also indicates that different jellyfish species may have distinct differences in their toxin composition and biological activity. While both AnC and OnC demonstrated comparable PLA_2_ activity that significantly exceeded TeC, only OnC exhibited marked hemolytic activity. The reason may be that the phospholipases in AnC have substrate selectivity. It may also be that, due to the structural constraints of AnC phospholipases, optimal membrane interaction in murine systems was prevented [50,51].

Metalloproteinases represent a typical toxin family in the venoms of jellyfish and various other venomous organisms. They can induce hemorrhage and necrosis through modulation of coagulation factors, platelet function, fibrinolytic activity, and vascular integrity [44,52,53]. Our results revealed significantly stronger metalloproteinase activity in OnC than in AnC. Gelatin zymographic analysis demonstrated that while both AnC and OnC possessed gelatinolytic activity, distinct banding patterns were observed in AnC and OnC. The gelatinases mainly include MMP-2 (gelatinase A, 72 kDa) and MMP-9 (gelatinase B, 92 kDa), which are both key matrix metalloproteinase family members, and the band variability in the gel potentially attributes to their proenzyme/activated forms and post-translational modifications (e.g., glycosylation). Notably, the observed lytic bands at ~110 kDa for AnC and ~100 kDa for both OnC and TeC may reflect either glycosylated MMP-9 variants or the presence of additional MMP isoforms contributing to the collective gelatin degradation activity [38,54].

Serine proteases, characterized by their catalytically active serine residues, represent a toxin family capable of inducing coagulopathy, hemorrhage, and hemodynamic shock through interfering with the coagulation and fibrinogenolytic system [55,56,57]. Notably, fibrinogenolytic serine proteases, particularly those with plasmin-like activity, mediate fibrinogen degradation through multisite cleavage, generating soluble fibrin degradation products. This fibrinolytic activity exhibits functional synergy with metalloproteinases in venom systems [58]. Both AnC and OnC exhibit fibrinogenolytic activity mediated by serine protease, with the enzymatic activity of OnC being significantly higher than that of AnC, and these proteolytic effects can be effectively inhibited by the addition of 1,10-phenanthroline.

Cnidarian venoms have been reported to exert cytotoxic effects across diverse cell lineages. For example, the venom of *Rhopilema nomadica* showed dose-dependent cytotoxicity to human hepatocellular carcinoma (HepG2), human breast adenocarcinoma (MDA-MB-231), human normal fibroblast (HFB4), and human normal lung fibroblast (WI-38) cell lines [7], along with the cytotoxic activity of *Pelagia noctiluca*, *Phyllorhiza punctata*, and *Cassiopea andromeda* venoms toward L929 cells [59]. In this study, AnC failed to demonstrate significant cytotoxicity against all three tested normal cell lines and five tumor cell lines, whereas OnC exhibited concentration-dependent cytotoxicity in all evaluated cell lines except THP-1. As suspension cells, the microenvironment around THP-1 is different from those of adherent cells (such as some tumor cells) and semi-adherent cells (such as RAW 264.7). Therefore, compared with adherent cells and semi-adherent cells, the fluid dynamic characteristics around THP-1 cells may cause differences in the distribution and concentration of venom components when they approach the cell surface, which may lead to a decrease in the toxic effect. To sum up, these findings are consistent with the results of the demonstrated enzymatic activity profiles mentioned above, suggesting that OnC possesses substantially stronger toxic potential compared to AnC.

Morphological and biochemical analysis both demonstrated distinct functional specialization between these two nematocyst types. O-isorhizas, with their spiral-structured nematocyst threads, exhibited superior penetration and discharge capacity. Furthermore, OnC demonstrated significantly higher metalloprotease, serine protease, and hemolytic activities, suggesting its potential dominant role in the processes such as prey capture and defense, tissue degradation, coagulation disruption, and hemorrhage induction. In contrast, Anisorhizas is relatively small in size, with significantly weaker discharge capacity as well as metalloproteinase and serine protease activities, and the toxic effects and tissue damage induced by Anisorhizas may be milder. However, it may have preferentially enhanced other functions such as cooperative predation and balancing energy consumption. The synergistic effect among different types of nematocysts may be an optimized predatory strategy, balancing immediate attack (O-isorhizas) with sustained metabolic efficiency (Anisorhizas).

In addition to their toxic effects, jellyfish venoms exhibit multiple biological activities, including antioxidant activity, antiarrhythmic activity and antimicrobial properties. Antimicrobial peptides (AMPs) such as aurelin, CgDef, and tregencin A/B have been identified in various jellyfish species including *Aurelia aurita*, *Chrysaora quinquecirrha*, *Carybdea marsupialis*, and *Rhopilema esculentum* [40,41,42,43,60]. Notably, for the 10 tested pathogenic bacteria, AnC and OnC exhibited significant inhibition against *Vibrios mimicus*, a clinically relevant pathogen causing gastroenteritis and cholera-like diarrhea characterized by abdominal pain, diarrhea and vomiting [61]. These results indicate that the antimicrobial effects of AnC and OnC are likely related to the unique cell wall structure or the metabolic pathways of pathogenic bacteria. Further studies on their antimicrobial spectrums and molecular mechanisms of the antimicrobial effects may provide new leads for marine drug discovery.

Previous studies by Kazuki et al. [62] reported a relatively low antioxidant activity in *N. nomurai* umbrella tissue (0.00541 μM trolox equivalent (TE)/mg by ORAC assay), whereas our investigation demonstrated significantly higher antioxidant activity for nematocyst extracts (AnC 209.7 ± 16.99 μM TE/mg; OnC 116.0 ± 6.07 μM TE/mg by ORAC assay), suggesting that nematocysts may have evolved enhanced antioxidant systems to protect their venom integrity, maintain symbiotic relationships, and counteract environmental oxidative stress. Suganthi et al. [63] isolated Frc-3 and Fre-3 from the venom of *Chrysaora quinquecirrha*, with Frc-3 demonstrating scavenging activity against hydroxyl radicals (·OH) and nitric oxide radicals (NO·). Fre-3 also exhibited potent antioxidant properties, effectively eliminating DPPH radicals, superoxide anions (O^2−^), and ·OH [64]. Nisa et al. [65] extracted venoms from the nematocysts of five species of *Chrysaora* jellyfish, and all the venoms showed strong ABTS radical scavenging activities. Therefore, combined with our research, these results demonstrate the great ABTS radical scavenging potential of jellyfish venoms. Our findings also indicate that the catalase activity of AnC (89.05 U/mg) was 2.09-fold higher than that of OnC (42.48 U/mg), demonstrating its superior enzymatic antioxidant capacity. In short, AnC exhibits superior oxygen radical scavenging capacity and catalase activity compared to OnC. The potent antioxidant activity of Anisorhizas nematocysts likely represents a multifunctional adaptive mechanism that not only preserves venom efficacy and structural integrity but may also participate in modulating microenvironmental redox homeostasis and environmental stress adaptation.

Jellyfish venom induces cutaneous inflammatory responses, with potential systemic toxicity when entering circulation, though the molecular mechanisms underlying these effects remain poorly characterized [66,67,68,69,70,71,72,73]. This study provides the first comparative analysis of inflammatory responses induced by isolated monomorphic nematocysts. The results revealed that AnC exhibited significantly greater proinflammatory activity than OnC, as evidenced by its capacity to markedly elevate key inflammatory mediators. These findings suggest that AnC may contain special inflammatory components capable of specific immune receptor recognition and subsequent activation of proinflammatory signaling pathways. It potentially plays a role in the inflammatory rection of jellyfish stings. However, OnC may lack such proinflammatory components with high-affinity, or may not be able to effectively trigger such reactions due to structural differences. The specific proinflammatory molecules in AnC and their mechanisms need to be further analyzed in depth through component research.

## 4. Materials and Methods

### 4.1. Chemicals and Reagents

Percoll was purchased from GE Healthcare (Chicago, IL, USA). L929, H9c2, HaCaT, A431, THP-1, and RAW 264.7 cells were purchased from Shanghai Anwei Biological Technology Co., Ltd. (Shanghai, China). HepG2 and HCT116 cells were purchased from American Type Culture Collection (ATCC, Manassas, VA, USA). A Cell Counting Kit-8 (CCK8) was purchased from Dojindo Molecular Technologies Inc. (Kumamoto, Japan). Azocasein, 4-nitro-3-octanoyloxybenzoic acid (NOBA) and lipopolysaccharide were purchased from Sigma-Aldrich (St. Louis, MO, USA). *Bacillus subtilis*, *Staphylococcus aureus*, *Pseudomonas aeruginosa*, *Escherichia coli*, *Vibrio vulnificus*, *Vibrio mimicus*, *Vibrio natriegens*, *Vibrio alginolyticus*, *Vibrio parahaemolyticus* and *Vibrio anguillarum* were purchased from Guangzhou Lige Biotechnology Co., Ltd. (Guangzhou, China). ORAC assay kit was purchased from Abcam plc (Cambridge, UK). Catalase assay kit and NO assay kit were purchased from Beyotime Biotechnology Co., Ltd. (Shanghai, China). Mouse IL-1β ELISA kit and mouse TNF-α ELISA kit were purchased from UPPBIO Co., Ltd. (Wuhan, China). All the other reagents used were of analytical grade. Statistical analysis and graphing were performed using GraphPad Prism (version 9.3.1, GraphPad Software, San Diego, CA, USA). Schematic drawing was performed using FigDraw (www.figdraw.com, accessed on 23 August 2025).

### 4.2. Jellyfish Collection

Specimens of the jellyfish *N. nomurai* were collected from Laoshan Bay in Qingdao, China, in August 2023. The jellyfish tentacles were excised immediately using a method described previously [14]. The isolated tentacles were placed in plastic bags with dry ice and immediately transported to the laboratory, where the samples were stored in a −80 °C freezer until use.

### 4.3. Isolation and Purification of Two Monomorphic Nematocysts

**Figure 11:**
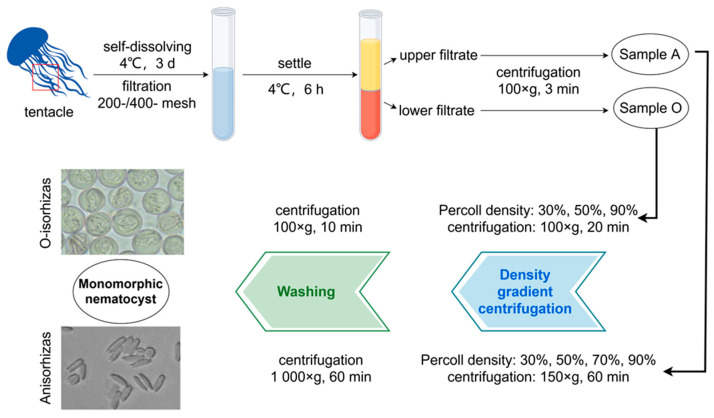
Flow chart of isolation and purification of two nematocysts from *N. nomurai* tentacles.

Figure 11 illustrates the isolation and purification protocol for monomorphic nematocysts from *N. nomurai*. Tentacle tissues (6 kg) were homogenized with high-Mg^2+^ artificial seawater (1:1.5 *w*/*v*, 0.6 M Mg^2+^, MgCl_2_·6H_2_O) and subjected to 3-day autolysis at 4 °C in a chromatography cabinet to maintain nematocyst stability, with bi-daily agitation (15 min/session). The lysate was sequentially filtered through 200- and 400-mesh sieves, followed by 6 h static sedimentation in the cabinet. The resulting biphasic solution was separated into upper (sample A) and lower (sample O) fractions for collection.

#### 4.3.1. Isolation and Purification of Anisorhizas

The sample A suspension was centrifuged at 100× *g* for 3 min at 4 °C. The resulting pellet was resuspended in high-Mg^2+^ artificial seawater (1:2 *w*/*v*, MgCl_2_·6H_2_O) and stored at 4 °C. A discontinuous Percoll gradient was prepared by sequentially layering 2 mL each of 90%, 70%, 50%, and 30% Percoll solutions (pre-chilled at 4 °C) in 15 mL centrifuge tubes, either by careful pipetting along the tube wall or by low-speed centrifugation to establish distinct interphase boundaries. The resuspended sample (2 mL) was then carefully loaded onto the gradient and centrifuged at 150× *g* for 60 min at 4 °C. The white suspension collected from the 50–70% interface was diluted with 10 volumes of high-Mg^2+^ artificial seawater and centrifuged at 1000× *g* for 60 min at 4 °C. This washing procedure was repeated 3–5 times to completely remove Percoll, yielding undischarged Anisorhizas nematocysts from *N. nomurai*.

#### 4.3.2. Isolation and Purification of O-Isorhizas

The Sample O suspension was subjected to primary centrifugation (100× *g*, 3 min, 4 °C), with the resulting pellet resuspended in high-Mg^2+^ artificial seawater (1:2 *w*/*v*, MgCl_2_·6H_2_O) and stored at 4 °C. A discontinuous Percoll density gradient was prepared in 15 mL centrifuge tubes by sequential layering of 90%, 50%, and 30% Percoll solutions. The resuspended sample (2 mL) was loaded onto the gradient and centrifuged (100× *g*, 20 min, 4 °C). The bottom-layer pellet was collected and washed through 3–5 cycles of resuspension in 10-fold volume of high-Mg^2+^ artificial seawater followed by centrifugation (100× *g*, 10 min, 4 °C), ultimately yielding undischarged O-isorhizas nematocysts from *N. nomurai*.

### 4.4. Nematocyst Content Extraction and SDS-PAGE Analysis

The contents from Anisorhizas and O-isorhizas were extracted using the method by Li et al. [74], with a slight modification. Briefly, the two nematocysts were suspended in deionized water and then ultrasonicated using a Misonix Sonicator (S-4000-010, Qsonica LLC, Newton, CT, USA). The sonication protocol was set as follows: 10 s working duration alternated with 20 s intervals, at a power output of 400 W. Each processing cycle repeated for 10 cycles. The treatment was terminated when microscopic examination confirmed that over 90% of the nematocysts had either discharged or ruptured. After centrifugation at 1000× *g* for 15 min at 4 °C, the supernatant was then dialyzed against phosphate-buffered saline (PBS, 0.01 mol/L, pH 7.4) for 8 h before use. The contents of two nematocysts were named AnC (total protein amount 3.3 mg; 0.55 mg protein/kg tentacle tissue) and OnC (total protein amount 8.4 mg; 1.4 mg protein/kg tentacle tissue), respectively, and the protein concentration of AnC and OnC was determined by BCA method. Then, 10 μg total protein of AnC and OnC were loaded onto 12% reducing sodium dodecyl sulfate (SDS)-polyacrylamide gels. Electrophoresis was performed according to the methods described by Wang et al. [14] using a PowerPac Universal Power Supply system (Bio-Rad, CA, USA). Protein bands were visualized with Coomassie brilliant blue staining.

### 4.5. Assessment of Nematocyst Discharge Capacity

**Figure 12:**
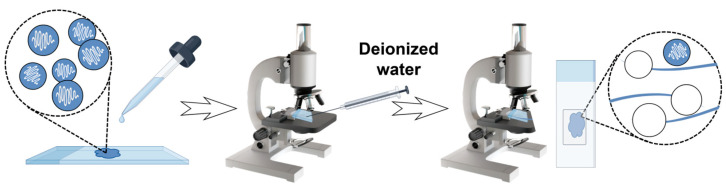
Schematic of the Anisorhizas and O-isorhizas nematocyst discharge assay procedure.

The discharge capacity of nematocysts was assessed using the method by Pyo et al. [3], with slight modifications. As shown in Figure 12. The purified Anisorhizas and O-isorhizas nematocyst pellets were resuspended in an appropriate volume of high-Mg^2+^ artificial seawater using gentle pipetting. A 10 μL aliquot of the nematocyst suspension was transferred to a microscope slide, and excess moisture was removed from around the coverslip edges with absorbent paper. After 1 min of air-drying to ensure surface adherence, a microscopic field was selected for observation. Using a microinjector, 2 μL of deionized water was introduced into the field, and nematocyst discharge events were immediately recorded under optical microscopy.

### 4.6. N. nomurai Tentacle Extraction

Tentacle extracted content (TeC) was extracted from *N. nomurai* tentacles (contain all types of nematocysts) according to the method described by Li et al. [74], with a slight modification. Briefly, the frozen tentacle tissue (5 kg) was thawed at 4 °C in artificial seawater (NaCl 28 g, MgCl_2_·6H_2_O 5 g, KCl 0.8 g, and CaCl_2_ 1.033 g, added distilled water to 1000 mL) at a quality and volume ratio of 1:1 for autolysis. After 4 d, the mixtures were filtered through a 400-mesh sieve to remove large tissue debris. The filtrate was then centrifuged at 500× *g* for 3 min at 4 °C, and sediments were washed three times with artificial seawater. The 50% and 90% Percoll solutions prepared with artificial seawater were placed at 2 mL per concentration from high to low in a 15 mL centrifuge tube, and then, the washed sediments were loaded at 2 mL. The centrifuge tube was centrifuged horizontally at 1000× *g* for 15 min at 4 °C, and then, the white sediments at the bottom were collected and washed three times with artificial seawater to obtain nematocysts containing venom. The sediments were subsequently subjected to ultrasonication for content extraction, as described in Method 4.4. A total of 10.12 mg of TeC was obtained (2.02 mg protein/kg tentacle tissue) and was frozen at −80 °C until use.

### 4.7. Cytotoxicity Assays

Cell viability was measured by the Cell Counting Kit-8 assay as previously described [75]. Three types of cells were involved: adherent cells (L929, H9C2, HaCaT, A431, HepG2, HCT116), semi-adherent cells (RAW 264.7), and suspension cells (THP-1). Cells were cultured in incubator at 37 °C with 5% CO_2_ until they reached 80% confluence. The dissociation protocol required 0.25% trypsin treatment only for adherent cells; for semi-adherent and suspension cultures, gentle pipetting sufficed for dissociation. Cell suspensions were seeded in 96-well plates at densities of 5000–12,000 cells/well (100 µL/well), followed by 24 h incubation. After medium removal, cells were treated with 100 µL of serially diluted AnC, OnC, and TeC in basal medium, with PBS and basal medium serving as negative and blank controls, respectively. Following 4 h treatment, 10% (*v*/*v*) CCK-8 reagent was added to each well for 1–3 h. Absorbance at 450 nm was measured using a microplate reader, with triplicate wells per condition. Cell viability was calculated as: [(OD_450_ treatment − OD_450_ blank)/(OD_450_ control − OD_450_ blank)] × 100%.

### 4.8. Enzyme Activity Assays

#### 4.8.1. Protease Activity Assay Using Azocasein

Protease activity was measured using a previously described azocasein-based assay [14]. Briefly, 50 mg of azocasein was accurately weighed and dissolved in 10 mL of buffer solution (50 mmol/L Tris-HCl, pH 8.0, containing 100 mmol/L NaCl and 5 mmol/L CaCl_2_) with gentle stirring to prepare a clear 5 mg/mL azocasein reaction solution. Then, 15 μL of AnC (stock solution, 1.32 mg/mL), OnC (stock solution, 2.88 mg/mL) and TeC (stock solution, 2.53 mg/mL) was mixed with 85 μL of the reaction solution, respectively, while Tris-HCl buffer served as the negative control. After thorough mixing, the samples were incubated at 37 °C for 1 h. The reaction was terminated by adding 200 μL of 5% (*w*/*v*) trichloroacetic acid (TCA), followed by incubation at room temperature for 30 min. The mixture was then centrifuged at 10,000 rpm for 20 min at 4 °C, and 150 μL of the supernatant was transferred to a 96-well plate. An equal volume of 0.5 M NaOH was added to each well to neutralize residual TCA. The absorbance was determined at 450 nm using a microplate reader. One unit of proteolytic activity was defined as an increase of 0.01 unit of absorbance at 450 nm, and the specific activity was expressed in units per milligram of protein (U/mg).

#### 4.8.2. Zymography of Proteases

The protease activity of AnC and OnC were examined according to the method by Li et al. [74], with minor modifications. Gelatin was purchased from Aladdin Biochemical Technology Co., Ltd. (Shanghai, China). Briefly, Gelatin (2 mg/mL) was dissolved in 20 mM sodium phosphate buffer (pH 7.4) and copolymerized with 10% polyacrylamide to prepare zymography gels. AnC (stock solution, 1.32 mg/mL), OnC (stock solution, 2.88 mg/mL) and TeC (stock solution, 2.53 mg/mL) were prepared in nonreducing sample buffer and then run on gels at 150 V/gel for 1 h at 4 °C. After electrophoresis, the gels were washed for 30 min twice with renaturing buffer (2.5% Triton X-100, 50 mM Tris-HCl, 5 mM CaCl_2_, 1 μM ZnCl_2_, pH 7. 6) and incubated for an additional 18 h at 37 °C for enzymatic reaction in zymography reaction buffer (50 mM Tris-HCl, pH 7.5, 5 mM CaCl_2_, 1 μM ZnCl_2_, 0.02% Brij-35). The gel was then stained with Coomassie staining solution. Clear zones in the gel indicate regions of proteolytic activity.

#### 4.8.3. PLA_2_ Activity

PLA_2_ activity was measured using a previously described assay [14]. Briefly, 25 μL of AnC (stock solution, 1.32 mg/mL), OnC (stock solution, 2.88 mg/mL) and TeC (stock solution, 2.53 mg/mL) was added to a 96-well plate, followed by 250 μL of reaction buffer (10 mmol/L Tris-HCl, pH 8.0, containing 10 mmol/L CaCl_2_ and 100 mmol/L NaCl), with Tris-HCl buffer serving as the negative control. Subsequently, 25 μL of NOBA was added, and the mixture was thoroughly mixed and incubated at 37 °C for 20 min. The reaction was terminated by adding 200 μL of Triton X-100 in an ice bath. Absorbance at 425 nm was measured using a microplate reader, with each sample analyzed in triplicate. An increase of 0.1 absorbance units (AU) at 425 nm corresponds to the release of 25.8 nmol of the chromophore 3-hydro-4-nitrobenzoic acid. PLA_2_ specific activity was expressed as U/mg.

#### 4.8.4. Fibrinogenolytic Assay

Fibrinogenolytic activity was measured according to the method by Bae et al. [58], with minor modifications, AnC, OnC, and TeC (100 μg/mL) were mixed with fibrinogen at a 10:1 ratio (sample:substrate), respectively. For inhibitor groups, samples were pre-incubated with 1,10-phenanthroline (10:1:10 ratio of sample: substrate: inhibitor), while fibrinogen alone served as the negative control. All reaction mixtures were incubated at 37 °C for 16 h. Following incubation, samples were mixed with loading buffer, boiled at 100 °C for 5 min, and analyzed by SDS-polyacrylamide gel electrophoresis. The extent of fibrinogen degradation or residual substrate levels indirectly reflects serine protease activity.

### 4.9. Hemolysis Assays

Hemolytic activity was evaluated according to the method by Li et al. [74], with minor modifications. In brief, erythrocytes from male ICR mice (30 ± 1 g body weight) were collected. Whole blood was collected via retro-orbital bleeding and mixed with PBS, followed by centrifugation at 1000× *g* for 10 min at 4 °C. After removing the serum and leukocyte layers, the washing procedure was repeated 2–3 times. Subsequently, 200 μL of the erythrocyte suspension was gently mixed with 9.8 mL PBS to prepare a 2% erythrocyte suspension, which was stored at 4 °C for subsequent experiments. For the assay, 100 μL of the erythrocyte suspension was mixed with an equal volume of AnC, OnC, or TeC at various concentrations (0.1, 0.4, 1.6, 6.4, 25.6, 51.2, 81.92, 102.4, and 204.8 μg/mL). PBS and Triton X-100 served as the negative and positive controls, respectively. The mixtures were incubated at 37 °C for 30 min, followed by centrifugation at 1500× *g* for 10 min at 4 °C. The supernatant (100 μL) was transferred to a 96-well plate (triplicate wells per sample), and absorbance at 540 nm was measured using a microplate reader. Hemolysis was calculated as: [(OD_540_ treatment − OD_540_ negative)/(OD_540_ positive − OD_540_ negative)] × 100%. The HC_50_ value indicates the concentration at which the sample induced 50% of the hemolytic effect achieved by the positive control (Triton X-100). The complete hemolysis concentration represents the concentration at which hemolysis effects was equivalent to the hemolytic effect induced by the positive control. All data were processed using GraphPad Prism 9.0 software.

### 4.10. Antimicrobial Activity

Antimicrobial activity was measured according to the method by Liu et al. [43], with minor modification. All procedures were performed under sterile conditions in a biosafety cabinet. The frozen bacterial strains were first revived and then inoculated into appropriate liquid medium, followed by incubation in a constant temperature incubator for 12–36 h until reaching the logarithmic growth phase (OD600 ≈ 0.6–1). For the assay, 20 μL of the logarithmic-phase bacterial suspension was mixed with 20 μL of the tested samples (AnC 1.32 mg/mL, OnC 2.88 mg/mL, and TeC 2.53 mg/mL) in 100 μL of liquid medium, while a control group was prepared by mixing bacterial suspension with an equal volume of PBS buffer. The inoculated culture systems were then continuously cultured under appropriate conditions in a shaking incubator. The optical density at 600 nm (OD600) was measured at 0 h, 4 h, 8 h, 12 h, and 16 h time points to monitor bacterial growth. All data were processed using GraphPad Prism 9.0 software. Differences in growth kinetics among groups were analyzed and compared through nonlinear regression analysis.

### 4.11. Antioxidant Activity Assay

#### 4.11.1. ABTS^+^ Scavenging Assay

Total Antioxidant Capacity (T-AOC) Assay Kit (ABTS Method, Microplate) (#D799298-0100) was purchased from Sangon Biotech (Shanghai) Co., Ltd. (Shanghai, China). The ABTS radical cation (ABTS^+^) scavenging assay was performed strictly according to the manufacturer’s protocol. The working solution was prepared by adding 7 mL of Reagent 1 to one vial of Reagent 2, followed by vigorous vortex mixing for 20 min and subsequent equilibration at room temperature. For the assay, the blank control consisted of 10 μL extraction buffer mixed with 190 μL working solution, while the sample groups contained 10 μL sample (1 mg/mL) mixed with 190 μL working solution. After thorough mixing and 20 min incubation at room temperature, the absorbance at 734 nm was measured with triplicate determinations for each group. The ABTS^+^ scavenging activity was expressed as % absorbance.

#### 4.11.2. Oxygen Radical Absorbance Capacity

The oxygen radical absorbance capacity of AnC and OnC quantified by ORAC method was performed as previously described, with slight modification [76]. In brief, 25 μL each of sample (AnC 1.32 mg/mL, OnC 2.88 mg/mL, and TeC 2.53 mg/mL), various concentrations of trolox standard solution (for construction of a standard curve) or blank buffer (as a control) were placed in the individual wells of a 96-well transparent microplate. Fluorescein working solution (150 μL) was added and the wells were agitated at 37 °C for 30 min. Subsequently, 25 μL of AAPH solution was added to each of the wells to initiate the reaction. The total volume of each reaction solution was 200 μL. The fluorescence intensity [480 nm (extraction)/520 nm (emission)] was then measured every 5 min over 60 min at 37 °C. As the reaction progressed, fluorescenin was consumed and the fluorescence intensity decreased. The inhibition of fluorescence decay was taken to indicate the presence of an antioxidant. The area under the kinetic curve (AUC) of the standards and samples was calculated as follows:

$$AUC=1+\frac{{RFU}_{1}}{{RFU}_{0}}+\frac{{RFU}_{2}}{{RFU}_{0}}+\frac{{RFU}_{3}}{{RFU}_{0}}+\cdots +\frac{{RFU}_{59}}{{RFU}_{0}}+\frac{{RFU}_{60}}{{RFU}_{0}}$$

The ORAC value, expressed as μM Trolox equivalents per milligram of protein (μM TE/mg protein), was obtained by normalizing the measured antioxidant activity to the protein content of each sample, representing its peroxyl radical scavenging capacity [59].

#### 4.11.3. Catalase Activity Assay

The catalase (CAT) activity was measured using a commercial Catalase Assay Kit (#S0051, Beyotime Biotechnology Co., Ltd., Shanghai, China) following the manufacturer’s instructions. Briefly, 3 μL of AnC, OnC, or TeC (1 mg/mL) was added to 1.5 mL microcentrifuge tubes, respectively, followed by the addition of assay buffer to a final volume of 40 μL, while the blank control contained only 40 μL assay buffer. After thorough mixing, 10 μL of 250 mM hydrogen peroxide solution was added and immediately mixed by pipetting. The reaction proceeded at 25 °C for 3 min before termination with 450 μL stop solution. Subsequently, 40 μL assay buffer was aliquoted into new 1.5 mL tubes, followed by addition of 10 μL of the terminated reaction mixture. After mixing, 10 μL of this solution was transferred to a 96-well microplate and mixed with 200 μL chromogenic working solution. Following 15 min incubation at 25 °C, the absorbance at 520 nm was measured with triplicate determinations for each sample. Catalase activity was calculated as: Catalase activity = (μmol hydrogen peroxide consumed × dilution factor)/(reaction time in minutes × sample volume × protein concentration), expressed in standard enzyme activity units.

### 4.12. Nitric Oxide Measurement and Cytokine Assay

#### 4.12.1. Nitric Oxide Measurement

The nitric oxide concentrations were quantified according to a previously reported method [68], with slight modifications. In brief, RAW 264.7 cells (1 × 10^4^ cells/well) were seeded in a 48-well culture plate and cultured for 24 h, treated with AnC, OnC and TeC (0.1 μg/mL) or LPS (1 μg/mL) and incubated for 24 h. NO concentrations in medium were determined using a Griess assay, which is an indirect measure of potential anti-inflammatory activity; Griess reagent (50 μL) was added to media supernatants (50 μL) and then incubated at 37 °C for 20 min in the dark. Absorbance was measured at 520 nm. NO concentrations were determined using 0–100 μM sodium nitrite standards.

#### 4.12.2. Cytokine Assay

The concentrations of TNF-α and IL-1β were quantified using commercial sandwich ELISA kits specific for mouse TNF-α (Catalog number SYP-M0036, UPPBIO Co., Ltd., Wuhan, China) and IL-1β (Catalog number SYP-M0026, UPPBIO Co., Ltd., Wuhan, China), respectively, in accordance with the manufacturers’ protocols. RAW 264.7 were cultured and treated as described in Method 4.12.1. Following treatment, the culture supernatants were collected for determination of cytokine concentrations.

## 5. Conclusions

This study successfully established the isolation and purification processes for two distinct nematocyst types (Anisorhizas and O-isorhizas) from *N. nomurai*. Comprehensive in vitro characterization revealed that O-isorhizas demonstrated potent discharge capacity, cytotoxic, hemolytic, metalloproteinase, serine protease, and antimicrobial activities, suggesting their primary role in venom delivery and tissue damage during envenomation. In contrast, the smaller Anisorhizas exhibited attenuated discharge capacity and toxicity, but enhanced antioxidant capacity, selective antimicrobial effects, and significant proinflammatory activity, indicating specialization toward complementary functions including cooperative predation and environmental stress adaptation. This study provides preliminary insights into the structure-function correlation between nematocyst morphology and biological activity, while providing a foundation for future investigations of compositional and functional diversity of nematocysts and mechanisms of jellyfish envenomation.

## Data Availability

The authors declare that all relevant data supporting the findings of this study are available within the article.

## Abbreviations

The following abbreviations are used in this manuscript:

AnC	Anisorhizas nematocyst content
OnC	O-isorhizas nematocyst content
TeC	Tentacle extract content
NOBA	4-nitro-3-octanoyloxybenzoic acid
T-AOC	Total Antioxidant Capacity
GSH	Glutathione
ORAC	Oxygen radical absorbance capacity
AMPs	Antimicrobial peptides
TE	Trolox equivalent
CAT	Catalase
